# Predation Risk Perception, Food Density and Conspecific Cues Shape Foraging Decisions in a Tropical Lizard

**DOI:** 10.1371/journal.pone.0138016

**Published:** 2015-09-18

**Authors:** Maximilian Drakeley, Oriol Lapiedra, Jason J. Kolbe

**Affiliations:** 1 Faculty of Life Sciences, The University of Manchester, Manchester, United Kingdom; 2 Department of Biological Sciences, University of Rhode Island, Kingston, Rhode Island, United States of America; The Hebrew University of Jerusalem, ISRAEL

## Abstract

When foraging, animals can maximize their fitness if they are able to tailor their foraging decisions to current environmental conditions. When making foraging decisions, individuals need to assess the benefits of foraging while accounting for the potential risks of being captured by a predator. However, whether and how different factors interact to shape these decisions is not yet well understood, especially in individual foragers. Here we present a standardized set of manipulative field experiments in the form of foraging assays in the tropical lizard *Anolis cristatellus* in Puerto Rico. We presented male lizards with foraging opportunities to test how the presence of conspecifics, predation-risk perception, the abundance of food, and interactions among these factors determines the outcome of foraging decisions. In Experiment 1, anoles foraged faster when food was scarce and other conspecifics were present near the feeding tray, while they took longer to feed when food was abundant and when no conspecifics were present. These results suggest that foraging decisions in anoles are the result of a complex process in which individuals assess predation risk by using information from conspecific individuals while taking into account food abundance. In Experiment 2, a simulated increase in predation risk (i.e., distance to the feeding tray) confirmed the relevance of risk perception by showing that the use of available perches is strongly correlated with the latency to feed. We found Puerto Rican crested anoles integrate instantaneous ecological information about food abundance, conspecific activity and predation risk, and adjust their foraging behavior accordingly.

## Introduction

Animals increase their chance of survival and reproduction in the wild by successfully responding to the ecological challenges they encounter. These responses are often shaped by decision-making processes, in which animals integrate information to produce behavioral responses that maximize their fitness. Particularly relevant decisions take place during foraging, when most animals need to acquire resources while also having to avoid predation [1–6]. Foraging research has shown the complexity of such decision-making processes by investigating the fitness consequences of foraging decisions and predicting those decisions as a function of both ecological and social conditions [7–9]. This is because foraging decisions depend not only on intrinsic factors such as physiology, body condition, cognitive abilities, sex, ontogeny, or variation in animal personalities, but also on extrinsic factors like the type or availability of food, structural habitat, and other environmental characteristics (S1 Fig). Moreover, when assessing the costs and benefits of any foraging decision in their local environment, animals also need to anticipate the decisions of both potentially competing conspecifics and predators [7,10,11].

The foraging decision-making process can be seen as a trade-off between the benefits brought by the decision (i.e., energetic gain) and the potential costs (i.e., risk of predation or costs of not investing time and energy in other activities such as reproduction) [6,12]. For instance, risk-taking behavior, or the possibility that uncertain outcomes negatively affect fitness, can be an important source of selection [11,12]. However, while increased vigilance (i.e., an active behavior in which individuals obtain ecologically relevant information about potential predators and/or competitors) is beneficial for predator avoidance and survival, increasing the time spent being vigilant reduces feeding rates [13] because, as stated by Nonacs and Blumstein, ‘a hiding or fleeing animal is not a foraging animal’ [14]. Indeed, understanding the drivers of the foraging decision-making process is important because i) context-dependent foraging behavior adjustments have the potential to modify population dynamics [15] and ii) the ability to modulate decision-making after evaluating potential costs and benefits could be favored by natural selection [14]. However, there are important gaps in our knowledge about whether and how the interplay among several ecologically relevant factors alters the balance between costs and benefits driving this process.

In the present study, we focus on some of the most crucial factors determining decision making in the context of foraging: food abundance and its interaction with both the threat of predation and the presence of conspecifics. On one hand, the influence of predation risk on the foraging decisions of animals has been addressed in several experimental studies (e.g., [16–20]) and it has been shown that assessing the risk associated with nearby predators is advantageous (e.g., [21,22]). For example, Pérez-Tris et al. [18] found that feeding rate of lizards decreased when perceived risk of predation was high. Interestingly, such a decrease occurred only when food resources were abundant. This suggests that more research is needed to understand if the relative importance of predation risk is decreased when food is scarce.

On the other hand, the impact of the presence of conspecifics on the decision-making of territorial, non-gregarious foragers is not well understood. This is in part because experiments have traditionally been conducted with individual animals in captive environments. In these experiments, predation has been simulated and the possible influence of competition has been assessed indirectly by food shortage conditions that mimic the effects of increased competition (e.g., [18]). Indeed, the presence of conspecifics could increase competition for resources. However, conspecific activity could also be beneficial for an individual forager because it can provide ecologically relevant information. For instance, it could be that the presence of conspecifics facilitates finding food resources, as would happen in a producer-scrounger system (e.g. [23]). Conspecific activity could also provide information on the presence of predators or it may allow for decreased vigilance as has been shown for group foragers [24]. Consequently, the role of the presence of conspecifics needs to be integrated into field experiments to understand how they influence the tradeoff between foraging and predation risk.

Here we present a standardized set of manipulative field experiments in the form of feeding assays in male *Anolis cristatellus* in Puerto Rico. In these trials, we presented lizards with foraging opportunities to investigate how the abundance of food, predation-risk perception, presence of conspecifics and the interactions among these factors determine the outcome of foraging decisions. Because foraging speed is a strong determinant of foraging success [25], in each of these trials we assessed foraging speed by timing how long it took lizards to capture a prey item once it was presented (i.e., latency to feed). Specifically, in the first experiment we manipulated food abundance in an experimental feeding tray to assess whether and how this modifies the willingness of anoles to exploit the food resource. We controlled for a number of intrinsic traits of individuals (e.g., body size) as well as environmental factors (e.g., temperature, local density of males, and whether or not conspecifics approached or fed in the feeding tray) to assess if these factors influence the tradeoff between the costs and benefits of exploiting newly available food resources. In the second experiment, we evaluated foraging responses of lizards at an increased distance from the focal lizard to the prey, a surrogate for increased predation risk [26,27]. We predicted that lizards should increase the latency to feed and that this should be driven by increased use of intermediate perches (i.e., increased vigilance behavior) to minimize predation risk when greater distances to prey make them more exposed to potential risks.

## Methods

### Ethics statement for observational and field studies

This study was approved by the University of Rhode Island IACUC AN11-09-005 and was authorized by the Puerto Rico Departamento de Recursos Naturales y Ambientales.

### Study species

Puerto Rican crested anoles (*Anolis cristatellus*) are territorial, diurnal lizards native to Puerto Rico and the Virgin Islands [28]. They are sit-and-wait foragers, typically perching on trees up to 3 m high while observing their surroundings for conspecifics, predators, and prey, which they often capture on the ground [27]. *Anolis cristatellus* spend most of their time in survey posture—head down, forequarters lifted off the substrate, and hindlimbs extended backwards up the vertical tree trunk [27,29]. Anoles in survey posture are receptive to foraging and they decrease their use of this position when fed to satiation [30]. Most of the time individuals spend on the ground corresponds to foraging bouts; otherwise, these anoles tend to remain on their perches [27]. Most of their diet consists of arthropods [31] and they have been reported to take advantage of novel resources in modified environments; for example, they exploit artificial light to feed at night in suburbs [31,32].

Food availability has been suggested to limit populations in Puerto Rico [27]. Accordingly, Licht [33] found in a study during the summer breeding season that *A*. *cristatellus* were rarely satiated and most lizards had nearly empty stomachs, which led them to forage rapidly when they were offered mealworms. At the same time, Puerto Rican *A*. *cristatellus* respond to a variety of terrestrial and aerial predators with a range of anti-predatory behaviors [34]. Semi-arboreal anole species are more vulnerable to predation when leaving a perch to forage for prey on the ground. This is because they are more exposed and therefore more easily localized by predators [35] and farther from refuges. Consequently, although *A*. *cristatellus* frequently engages in communication by displaying a colorful dewlap [36], they spend the majority of their time perched motionless [37], either vigilant to predators and conspecifics or monitoring for foraging opportunities [27]. Moreover, in populations of *A*. *cristatellus*, it is easy to assess the density of conspecifics, to determine if conspecifics other than the focal individual approach the experimental food resource by direct observation, and to manipulate the amount of food available in each foraging event.

### Study site

Our study site was a native pine forest (*Pinus caribaea*) near Bosque EstataldePiñones in northeast Puerto Rico. The distribution of pine trees at the site was relatively uniform, and the area surrounding the target trees was flat and covered in dense pine needles, occasionally with shoots sprouting through, small rocks, or broken branches. This open understory was conducive to maintaining a direct line of sight between focal anoles and the food resource. Potential predators observed in the forest include the Puerto Rican Ground Lizard (*Ameiva exsul*) and Greater Antillean Grackle (*Quiscalus niger*). *Ameiva* species are predators of anoles [30,26]. Therefore, two trials where *A*. *exsul* was within 4 m of the experimental feeding tray were terminated.

### Pilot trials

Pilot trials were conducted prior to the main experimental procedures. The aim was to determine the conditions in which free-ranging adult male *A*. *cristatellus* on their perches would respond to and approach potential prey items presented in a feeding tray.

1. **Food resource**. We used mealworms (*Tenebrio molitor*) as the food resource presented to *A*. *cristatellus*. This decision was made based on previous studies that successfully used them for supplemental feeding of anoles in Puerto Rico [33]. Mealworms used in our experiments ranged in size from 12 mm to 20 mm, well within the size range of prey items for this species [31]. Mealworms were easily localized by anoles because they continuously crawled around trays during the experimental trials.
2. **Experimental feeding tray**. A brown, rectangular tray (14 cm x 20 cm) lined with brown cardboard was selected as the experimental feeding tray. The 5-cm depth of the tray prevented the mealworms from escaping during the experiments while they were still visible to perching anoles. Because some anoles showed a reluctance to enter the tray from ground level, we attached gently sloping cardboard ramps to each edge of the tray to allow them easier access onto the tray lip (see S2 Fig)
3. **Saturation study**. Pilot trials showed that anoles would forage in trays with as few as 1 mealworm and as many as 20 mealworms. Observations were conducted to determine the point at which anoles would stop feeding despite still having mealworms available to eat. Five was the maximum number of mealworms consumed in a 15-min period, although the majority of anoles stopped returning to the feeding tray after consuming their third mealworm. Based on this result, food densities of two, five, and ten mealworms were used for the first experiment. Two mealworms were selected instead of one to account for the possibility that a non-focal anole might fed from the experimental tray before the focal anole arrived, which was observed during the pilot trials.
4. **Distance to the feeding tray**. After a focal male *A*. *cristatellus* in survey posture was located, the experimental feeding tray was placed directly in front of the lizard at varying distances. A feeding tray with five mealworms was placed at various distances between 1 m and 13 m away from the focal anole in survey posture. The longest distance recorded for an adult male to approach and forage from the experimental tray was 11.7 m, and the success rate of anoles approaching the tray dropped below 50% when it was placed more than 7 m from the perch. Based on these results, a distance of 2–3 m was used for Experiment 1 to assess food abundance effects whereas a distance of 5–6 m was established for Experiment 2 to investigate the influence of perceived predation risk.
5. **Neophilia**. We assessed if anoles had an inherent tendency to explore new objects within their territories because such a tendency could represent a confounding factor when interpreting results of our experiments. This possibility was ruled out because anoles never approached an experimental feeding tray when it did not contain any mealworms (n = 10).

### Experimental protocol

All experiments were conducted on clear days in February and March 2013 between 1000–1600 h, when *A*. *cristatellus* are most active [33]. Male *A*. *cristatellus* were located perched on trunks or branches with their heads down in survey posture at heights of 30–270 cm. Only anoles perched in this foraging position were selected, as anoles facing upward may be satiated ([30] and see Results section below). The latency to feed was determined from videos as the time for the focal anole to retrieve a mealworm from the tray.

#### Experiment 1: Food abundance and the presence of conspecifics

The aim of this experiment was to assess how the foraging decisions of anoles are determined by food abundance. We also incorporated observational data on the presence of conspecifics interacting with the feeding tray to understand if their presence altered foraging decisions of focal individuals. A feeding tray was placed at 2.30 +/- 0.40 m (mean +/- s.d.) from the base of the tree upon which a focal anole was perched and in direct view of the anole. Two, five, or ten mealworms were randomly assigned to the trial and placed in the tray. To minimize the disturbance caused by placing the tray, it was first covered with a retractable cover tied to a string before it was placed. This cover concealed the mealworms from the view of the anole until the experimenter removed the cover from a distance (see S2 Fig). A 2-min habituation time was imposed before removing the cover, which marked the beginning of the experiment.

Trials were recorded using a Nikon D90 Digital SLR Camera with a zoom lens placed on a tripod approximately 1.5 m behind the tray. This captured a video of the tray, an image of the starting position of the anole on its perch, and the space between the perch and tray, allowing observation of all behaviors while providing a non-invasive method for determining original perch height. Time of day, temperature and humidity at the level of the feeding tray, initial perch height and perch diameter on the original tree, and the actual distance from the tray to the base of the tree were recorded. Throughout the experiment, a Dictaphone was used to record behavior of the focal anole, and the presence of non-focal conspecifics. A conspecific foraging interaction was recorded when other adult males either made advances towards the tray or foraged in the tray before the focal anole removed or consumed a prey item from the tray. Thus, the number of conspecifics approaching the feeding tray was not a manipulated factor in the experiment. Rather, we incorporated natural variation, which was similar for each treatment, allowing us to include this ecologically relevant factor in the analyses. If a focal anole did not approach the tray after 10 min from when the cover was removed, the experiment was ended and excluded from the analyses (n = 3). Finally, a visual encounter survey was conducted in a 7-m radius surrounding the tray to estimate the local density of other adult males, which was used as a surrogate for indirect, potential competition. Note this factor differs from conspecific foraging interaction in that it is a measure of local abundance independent of whether conspecifics approached the feeding tray or not. A minimum of 10 m was traversed before starting to search for other focal anoles for the next experimental trial. Therefore, all focal anoles used throughout the study were individuals that had not been observed in previous trials.

#### Experiment 2: Perceived predation risk and the presence of conspecifics

Further foraging assays were conducted to assess how perceived predation risk influences the latency to feed of anoles. We predicted an increase in vigilance behavior—as measured by the use of intermediate perches between the focal anole and feeding tray [27, 35]. Because anoles are most vulnerable to predation when they leave their perches [26,27], they should perceive an increased distance between their perch and their prey as a more risky situation. To assess how increased risk perception affects the latency to exploit a foraging opportunity, a foraging assay was conducted by doubling the distance to an experimental feeding tray containing five mealworms (i.e., distance of 5–6 m from the perch of the focal anole [n = 14]). This distance was used because a distance of 7 m yielded 50% foraging success in pilot trials and it was likely within the home range of *A*. *cristatellus* observed by Fitch et al. [31]. We recorded the number of perches along the route from the original perch location to the feeding tray. ‘Potential perches’ were trees and branches within 0.5 m of the line of sight directly between the original perch and the tray. ‘Actual perches’ were those used by focal anoles on the way to the tray. ‘Conspecific foraging interaction’ and previously described intrinsic and environmental variables were also measured during this assay. Experiment 2 was conducted concurrently with Experiment 1, making results from both experiments comparable.

#### Snout-vent-length estimation

Capturing individuals to measure their body size, measured by the standard snout-vent length (i.e., SVL) in lizards, entails two complications. First, the capture process can be invasive and may affect future behavior [38]. Second, the requirement to capture anoles in order to measure SVL means that individuals that escape cannot be used in data analysis. Here we developed a non-invasive technique to estimate SVL by using photographic images. First, a picture of the perching focal anole was taken at the beginning of the experiment. Then, at the end of the experiment, another picture was taken of a ruler held in the original position and orientation of the focal anole with the camera at the same position and focal length. One of each pair of photographs, usually the one with the ruler, was reduced to between 25% and 40% transparency and then pasted over the corresponding image using photo-editing software (Microsoft Digital Image Pro). The semi-transparent image was then resized and rotated until prominent features of the background, such as the edges of leaves or the branches of the tree were aligned (S2 Fig). In this way, it was possible to superimpose the image of the ruler over the image of the anole while maintaining the same scale for each image to estimate accurately the SVL. A measurement was taken from the tip of the snout to the center of the hip joint, where the vent is located on the underside of the anole. When the ruler was off to one side or the other, it was necessary to draw two parallel lines out to the snout and hip. Occasionally, the anole’s head was raised up from the tree and it was necessary to subscribe an arc from the anole’s snout to touch the ruler, as if it were lying flat against the tree (S2 Fig). To assess the reliability of our SVL estimation procedure, we blindly repeated the SVL estimation procedure from pictures of individuals from which we captured to obtain their actual SVL measurements (n = 14). We found a correlation of 0.89 (p < 0.0001) between SVL measurements obtained from the two procedures.

### Statistical analyses

A general linear model (GLM) was conducted for Experiment 1 by using the R platform [39]. We tested alternative models including the effects of two main factors and several covariates on the latency to forage from the experimental tray (in log-transformed seconds). Factors analyzed corresponded to food abundance (three levels of mealworm abundance) and the presence/absence of conspecifics interacting with the feeding tray. Covariates tested in our models included morphology (SVL), structural habitat (height and diameter of the perch where the focal lizard was found), local density of lizards around the tray, and weather conditions (temperature and humidity). Models tested included the main effects as well as predicted interactions among them. Model selection was done by choosing the model with a significantly lower AICc score. In Experiment 2, because the number of perches is not a truly continuous variable, we used Spearman correlations for indicative purposes to represent the association between increased latency to feed and the number of perches lizards used while approaching to the feeding tray. A Kruskal-Wallis test was conducted to compare latency to feed between anoles that adopted a survey posture after feeding to those that perched upwards [30]. Finally, t-tests were used to assess if latency to feed changed in the presence of conspecifics. Effect sizes are provided to quantify the amount of differences between groups. Specifically, partial eta-squared (η^2^_p_) were manually computed to estimate effect sizes from models including other independent variables. Similar to linear regression, η^2^_(p)_ * 100 is interpreted as the percent of variation explained by each factor. The magnitude of the effect size is interpreted as small (< 0.06), medium (0.06 < η^2^_p_ < 0.14) and large (<0.14). To report the effect size of the distance treatments on the latency to feed, we used the “cohen.d” function in “effsize” R package [40]; specifically, we report Hedge’s G (a modified version of Cohen’s ‘d’ more suitable for small sample sizes). Finally, we provide Pearson’s (r) and (R^2^) to interpret the measures of association from correlations and regressions respectively.

## Results

### Experiment 1. Food abundance and presence of conspecifics

**Fig 1:**
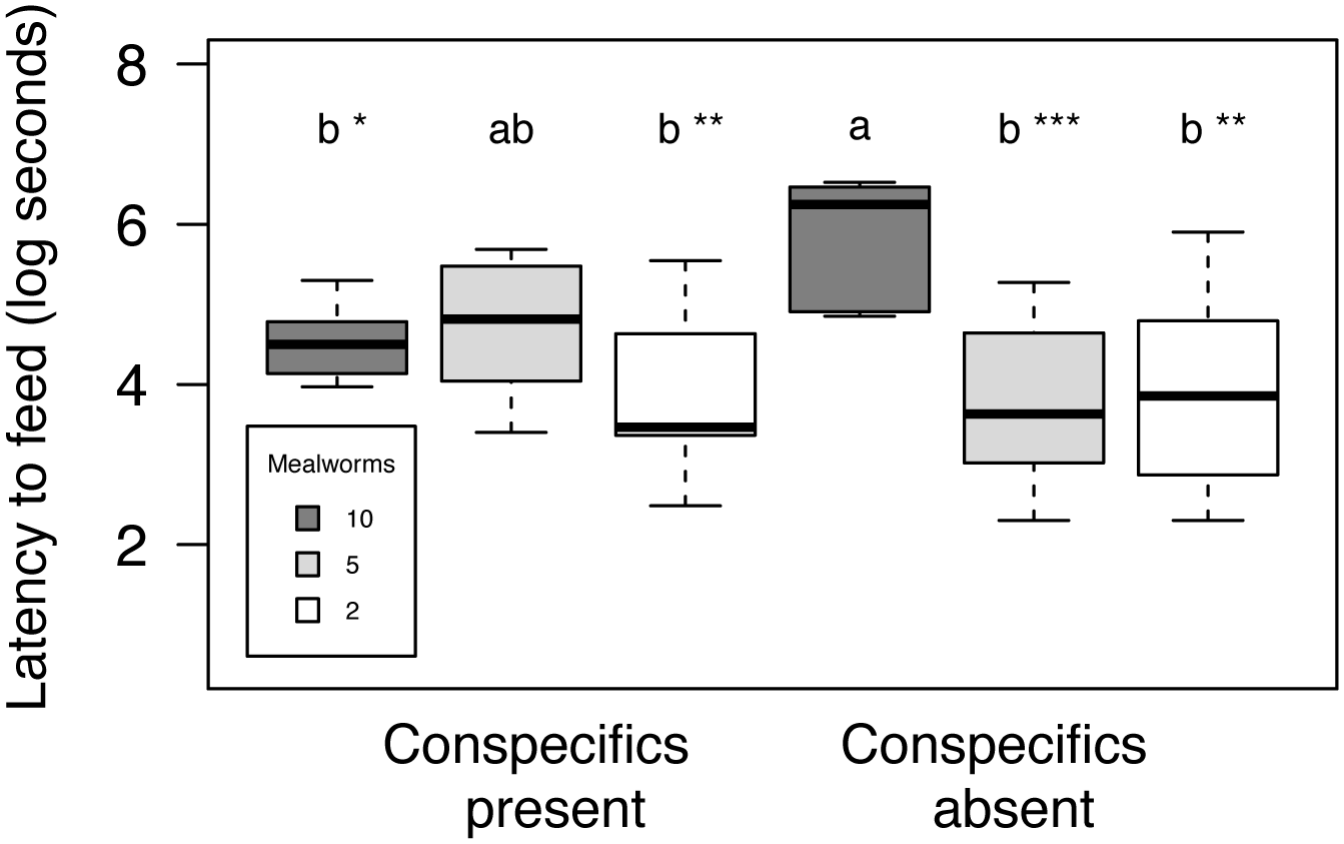
Latency to feed (log seconds) +/- SE for groups of individuals categorized according to the natural variation in presence or absence of conspecifics and three levels of experimentally manipulated food abundance (10, 5, and 2 mealworms). Comparisons among all six experimental treatments are provided. Letters “a” and “b” correspond to significantly different groups as analyzed using a Tukey's range test. Levels of significance are represented by ‘*’ (p < 0.10), ‘**’ (p < 0.05), and ‘***’ (p < 0.001).

The latency to feed from the experimental tray increased with the number of mealworms presented (Effect size (η^2^_p_) = 0.217; p < 0.01) and the presence of conspecifics interacting with the feeding tray (Effect size (η^2^_p_) = 0.051; p < 0.003) (see Table 1). We found a significant interaction (η^2^_p_ = 0.208; p < 0.05) between the presence of conspecifics at the feeding tray and the number of mealworms. That is, anoles showed longer latencies to forage when there was no conspecific present and the food abundance was highest (Fig 1). Irrespective of the interaction between food density and conspecific presence, larger males took less time to feed than smaller males (η^2^_p_ = 0.110; p = 0.008; Table 1). Alternative models fit the data poorly (AICc difference > 2). When included as covariates in the best fitting model (see Table 1), we did not find an association between temperature (η^2^_p_ = 0.004; p = 0.70), humidity (η^2^_p_ = 0.004; p = 0.71), perch height (η^2^_p_ = 0.086; p = 0.07), perch diameter (η^2^_p_ = 0.003; p = 0.74) or local density of conspecifics (η^2^_p_ = 0.011; p = 0.60) on the latency to feed from the experimental feeding tray for any of the experiments. Means and ranges for these measurements and SVL are detailed in S1 Table.

**Table 1:** Results for the best-fitting GLM model (AICc difference > 2 as compared with alternative models) describing latency to feed from the experimental feeding tray. Only factors significantly affecting latency to feed are included.

n = 44		Estimate	SE	t—value	p—value
	Intercept	9.98	1.99	5.01	<0.001
Factors					
	Conspecifics present	-1.67	0.51	-3.28	0.003
	Food abundance				
	2 mealworms	-1.93	0.49	-3.96	<0.001
	5 mealworms	-1.74	0.51	-3.44	0.001
	Conspecifics present * Food abundance				
	2 mealworms	1.72	0.72	2.4	0.021
	5 mealworms	2.13	0.75	2.85	0.007
Covariates					
	SVL	-0.69	0.32	-2.13	0.039

### Experiment 2. Perceived predation risk and presence of conspecifics

**Fig 2:**
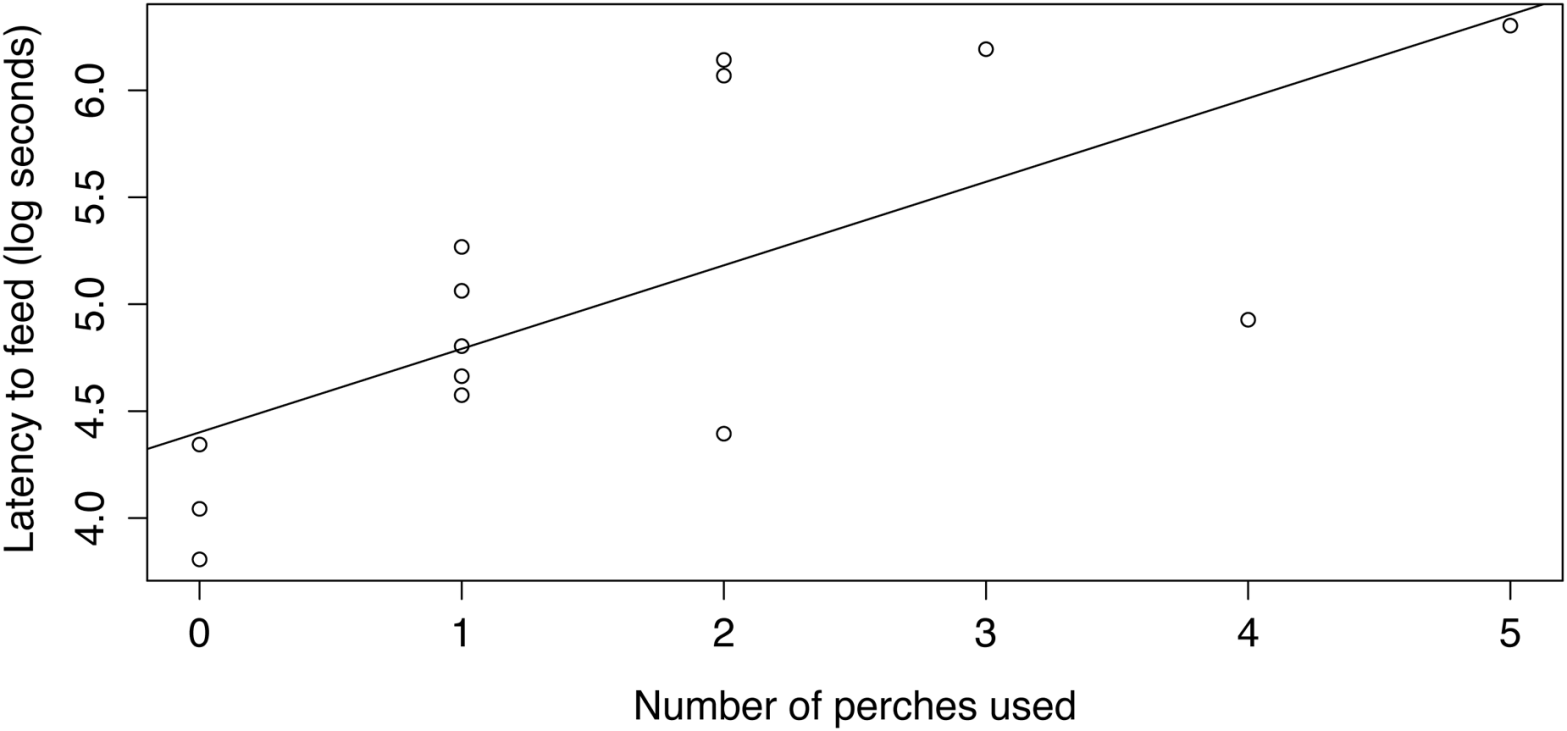
Latency to feed (log seconds) from an experimental feeding tray located 5 m from the focal lizard compared to the actual number of perches used while approaching the tray.

Latency to feed was longer at an increased distance from the perch to feeding tray (as compared with the same mealworm abundance at a shorter distance in Experiment 1; Hedge’s G = 0.879, p < 0.03). As predicted, the latency to forage increased in association with the number of perches used by lizards when approaching the feeding tray (Pearson correlation test: estimate = 0.495, p < 0.01; Fig 2), irrespective of their body size (Pearson correlation test: estimate = -0.251, p > 0.20). The use of secondary perches was largely associated with their availability (Pearson correlation test: estimate = 0.916; p < 0.001; S3 Fig). Interestingly, such a relationship between latency to forage and the number of perches used did not exist for the treatment with the same mealworm abundance (i.e., the five mealworm treatment) but at a shorter distance in Experiment 1 (Pearson correlation test: estimate = -0.120; p > 0.68; S4 Fig), suggesting this pattern arises only when perceived predation risk is high (i.e., only at longer distances). Finally, as opposed to results from Experiment 1, the latency to forage from the feeding tray did not depend on the presence of conspecifics (p = 0.412). Rather, time increased similarly with the use of perches irrespective of whether a conspecific approached the feeding tray or not (S5 Fig).

#### Survey posture and latency to forage

**Fig 3:**
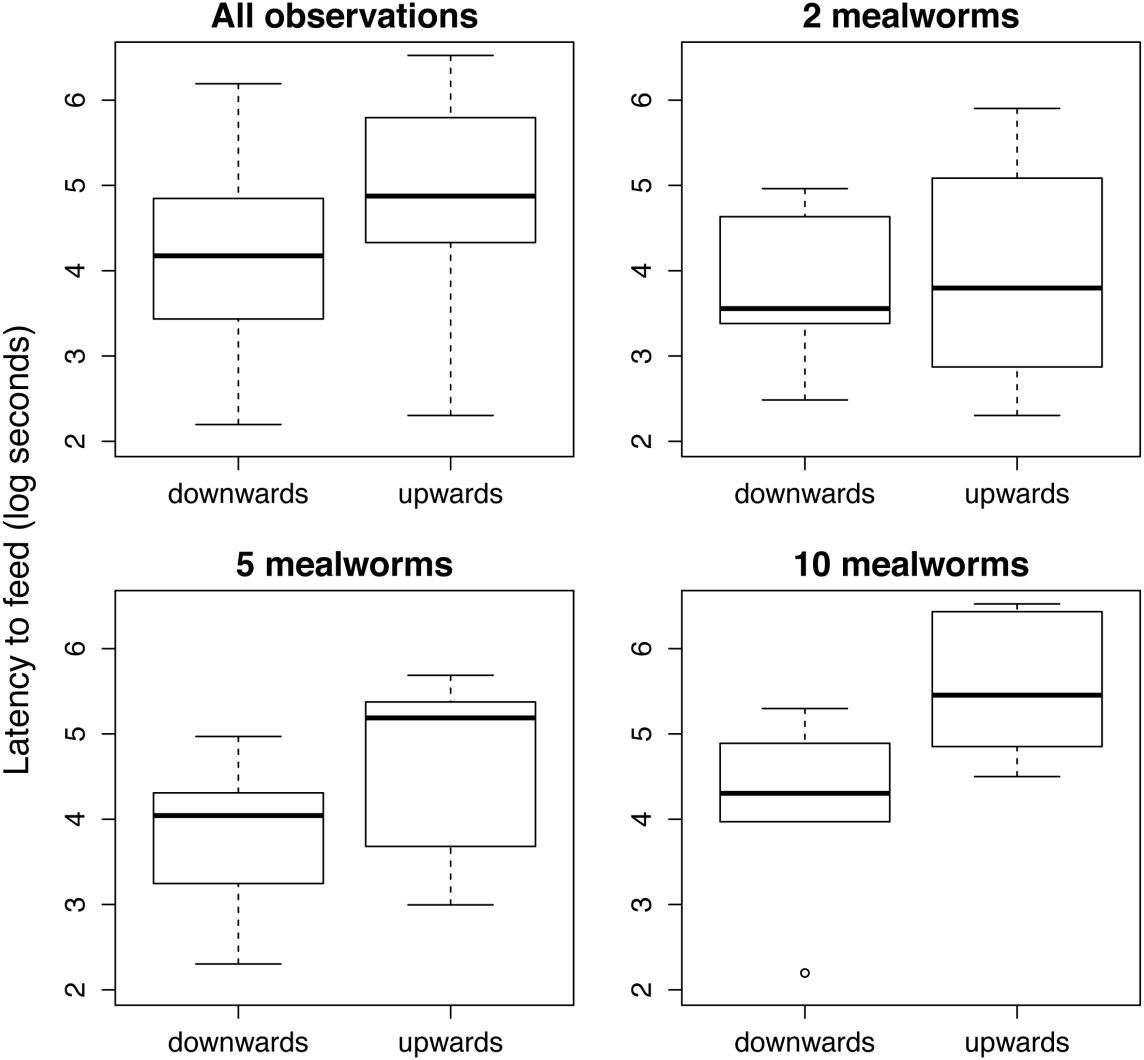
Upper left: Differences in the latency to feed (log-seconds) for lizards that perched upwards (non-survey posture) vs. downwards (survey posture) after foraging from the experimental tray and returning to a perch including all observations in Experiment 1 (n = 44). Upper right and lower left and right: latency to feed shown separately for the food abundance treatments (i.e. the number of mealworms presented in the experimental tray).

Individuals perched upwards at the end of the experiment had longer latencies to feed (mean ± SD: of 241 s ± 56 s vs. 77 s ± 17 s, Hedge’s G = -0.700, p < 0.01) (Fig 3, upper left) and tended to return fewer times to feed on additional mealworms (0.83 ± 0.11 vs. 0.62 ± 0.13 times, although this was not significant; Hedge’s G = 0.443 p = 0.23). This result suggests that these lizards might have been less motivated (i.e., not as hungry) at the start of the experimental procedure. Indeed, effect size estimation confirms that the pattern of higher latencies to forage in anoles perching upwards at the end of the experiment emerges only in the conditions where food was abundant [Hedge’s G = —0.083, p = 0.86 when we provided 2 mealworms; Hedge’s G = -0.723, p = 0.17 for 5 mealworms; and Hedge’s G = -1.380, p = 0.05 when 10 mealworms were presented] (Fig 3).

## Discussion

The willingness of Puerto Rican crested anoles to feed was determined by a decision-making process in which anoles integrate instantaneous ecological information and adjust their behavior accordingly. Crested anoles were more willing to approach a food resource when it was scarce compared to when it was abundant (with a magnitude of the effect size > 22%). Importantly, anoles were only significantly less willing to approach the feeding tray when mealworms were twice as abundant (i.e. 10 mealworms) as the threshold number on which they stopped feeding from the tray in a saturation pilot study. This suggests that the higher costs of losing a foraging opportunity when food is scarce are taken into account by foraging anoles. This is consistent with previous results showing that other lizards can assess the amount of food reward when making other ecologically relevant decisions. For instance, when being chased in simulated predation bouts, the flight distances of Balearic lizards (*Podarcis lilfordi*) were shorter when food was present [41] and they also emerged more rapidly from shelter when the food reward was higher [38]. Our study, however, suggests that the decision-making process in lizards is even more complex. Foraging anoles seem to take into account the behavior of nearby conspecifics before making a foraging decision. Consequently, anoles showed longer latency to feed in the absence of other conspecifics and when food was abundant (this interaction explained more than 21% of the total variance in the latency to feed, irrespective of other variables in the model). Therefore, although anoles tend to be more motivated to exploit a foraging opportunity when food is scarce, they will prioritize vigilance behavior when food is abundant and there are no conspecifics around. The fact that the presence of conspecifics spurred the response of individuals particularly when food was not abundant has several possible explanations. First, the presence of conspecifics could facilitate finding food resources, as happens in a producer-scrounger system [23]. However, under a producer-scrounger scenario we would expect focal lizards to be more willing to join if the food patch is large, which is not the pattern observed in the present study, in which latency is reduced when food is less abundant. Second, it could be that the presence of conspecifics represents increased intra-specific competition. Under this scenario, animals should perceive that the costs of not foraging are high due to decreased chances to improve their body condition. Although the absence of a relationship between the local density of lizards around the focal lizard and its latency to exploit a food resource in our experiments does not seem to support this hypothesis, we can not reject this possibility. Alternatively, the presence of conspecifics could be used as a cue of lower predation risk, thus enhancing foraging activity, as happens in group foraging animals [24]. Decreased foraging efficiency driven by indirect cues from predators has been shown in other animals, including lizards [42]. To our knowledge, the possibility that the foraging behavior of anoles is influenced by the decisions of other conspecifics has not been acknowledged before. However, our results are consistent with previous results in brown anoles (*Anolis sagrei)* suggesting they are less reactive to predation threats when a conspecific is present [43].

Our results showed no association between the latency to feed and local density of anoles, but an association with conspecifics approaching the feeding tray. Therefore, foraging decisions seem to be more importantly determined by instantaneous adjustments of foraging behavior to current conditions rather than by longer-term average conditions. This is in line with previous studies suggesting that anti-predator behavior is largely influenced by instantaneous ecological conditions (e.g., [19,44–46]) and implies that behavioral plasticity is important for determining the foraging behavior of *Anolis* lizards.

Larger animals—in terms of SVL—had lower latency to feed from the experimental tray (explaining more than 10% of the total variance in the latency to feed). Indeed, this could be because they are physically capable of moving faster [47]. However, the actual differences in the latency to feed are very small. Alternatively, it has been suggested that larger animals may be subjected to less predation risk [47], but we acknowledge this result could be the consequence of other unmeasured factors, such as differences in social dominance or different behavioral strategies among individuals differing in body size.

Motivation could be a confounding factor determining the willingness of individuals to take risks as well as influencing their performance [48–50]. We have not directly assessed motivation in our experimental procedure and, indeed, some of the individuals tested could be satiated while others could be starving and thus more willing to take risks. However, we do not expect that the mean motivation of individuals in each experimental treatment differed initially. Moreover, food density treatments were randomly assigned to individuals and there is no reason to expect that other conspecifics will be more or less willing to feed if the focal individual is more or less satiated. Importantly, we exclusively conducted experiments on individuals that were in ‘survey posture’ [27,29]. We show that survey posture is indirect evidence that individual anoles are motivated to forage (Fig 3). This supports the assumption that all individuals included in our study were willing to engage in foraging activities. However, we acknowledge this does not entirely rule out the existence of some degree of individual differences in motivation.

The influence of habitat structure on the existing tradeoff between anti-predator and foraging behaviour is poorly understood in terrestrial vertebrates [see 50]. Experiment 2 consisted of foraging assays conducted to assess the influence of increased perceived predation risk on vigilance behavior during foraging decisions. In these assays, time increased linearly with the number of perches used between the initial perch and the food tray. The latency to feed largely depended on the use of these perches. However, this did not happen when the feeding tray was located at a shorter distance. This suggests that a larger distance was indeed perceived as an increased predation risk [45], causing lizards to use more perches to assess the presence of predators. Moreover, the time to feed when the feeding tray was located at a longer distance was not influenced by the presence of conspecifics. This suggests that the perceived danger when animals need to cover a longer distance from their perch to the food resource is higher such that the presence of conspecifics is not taken as sufficient evidence of decreased predation risk. Therefore, although anoles take into account both costs and benefits to make foraging decisions, they may invoke a hierarchy in which predation risk outranks the benefits from foraging. Because being killed by a predator indeed decreases fitness, it seems reasonable to expect that prioritizing anti-predator behavior over foraging should be favored by natural selection in most circumstances [11].

Habitat structure can also heavily influence perceived predation risk [5]. This is because habitat structure determines the proximity and availability of shelters where potential prey can hide and/or assess the presence of potential predators [38,41]. Importantly, because anoles use pursuit-deterrent strategies to avoid predation [51], continuously assessing the presence of predators maximizes their chance of escaping using this strategy [5]. This is consistent with our findings that instantaneous ecological information is used by foraging crested anoles (e.g., the presence of foraging conspecifics). Such information is a more accurate indicator of predation risk than, for instance, the mean density of predators in an individual’s home range [5]. Indeed, the presence of a nearby shelter has been shown to influence foraging behavior in other lizard species [38,41]. Our finding that the availability of intermediate perches largely influences the latency to feed from the experimental tray is consistent with the prediction that habitat structure determines anti-predator behaviour in *Anolis* lizards (e.g., [44]).

Other factors might influence the decision making process (see S1 Fig). We attempted to rule out the most plausible alternative confounding factors. For instance, we did not find any effect of environmental factors such as temperature and humidity. This is not to say that temperature and humidity are not relevant factors influencing lizards’ foraging behavior. Indeed, the influence of weather conditions has been shown to determine spatial niche use by anoles [52] and anti-predator behavior in other lizards [53]. Rather, we show they do not exert any effect in the range of temperature and humidity in which our experiments were conducted, which were well within the range of normal activity for these lizards [54,55]. We also conducted pilot trials to reject the possibility that animals were spontaneously attracted to the objective irrespective of the presence of food (i.e. neophilia). Finally, we acknowledge that results could vary if foraging behavior of anoles varies temporally. Seasonal fat cycles have been associated with reproductive cycles in anoles [56] and other vertebrates [57]. We minimized the possibility that our results were driven by differences in the reproductive status because all our foraging assays were conducted at a time of year when reproduction in crested anoles is low [58,59]. The foraging behavior of anoles could also change at different times of the day if anole predators and/or competitors are active at specific times, and thus influence patterns of foraging activity at finer scales. For instance, Kolbe et al. [26] showed that daily patterns of perch height use varied in anole communities in association with the foraging activity of a terrestrial predator. Furthermore, the influence of the presence of predators on the foraging activity of *Anolis* species was also shown to interact with weather conditions [47].

In conclusion, our study suggests that the foraging behavior of anoles is a complex context-dependent process determined by both intrinsic and extrinsic factors. Foraging decision-making is affected by predation risk perception and resource availability [60,61] as well as by the presence of conspecifics approaching the feeding tray, but not by their local density. Foraging decisions are mainly governed by a trade-off between the benefits of acquiring food resources and the potential costs of a predation event [5,12]. Therefore, the ability of animals to adjust their behavior in ecological time by assessing the costs and benefits of making a foraging decision should be particularly relevant in ecological contexts of increased environmental uncertainty. Consequently, behavioral plasticity in the decision making process is expected to play a crucial role in determining population dynamics in the current context of human-induced rapid environmental changes such as the urbanization process or non-native species colonization of novel areas [62–64]. Indeed, predators have been suggested to represent strong selective forces in anoles and other lizards [46,65,66]. Consequently, the ability to collect, process and take advantage of information from conspecifics could be favored by natural selection [67], particularly if the use of such information minimizes the risk of predation. Altogether, this would imply that individual foragers acquire valuable ecological information by assessing the behavior of conspecifics.

## Supporting Information

S1 Fig Factors potentially influencing the foraging decision-making process when an animal encounters a foraging opportunity (in boxes). Dashed-line boxes indicate intrinsic factors and solid-line boxes are extrinsic factors. Arrows connecting factors represent the direction of the effects, which are both direct (solid lines) and indirect (dashed lines). Indirect effects influence the foraging decision by modifying an intermediate factor. Factors not assessed in this study are included here to provide a more complete conceptual framework for discussion.
(PDF)

S2 Fig Experimental feeding tray with cardboard ramps (left). The picture shows a male *A*. *cristatellus* consuming a mealworm. Also visible in the foreground is the retracted cover used to hide mealworms from view during the set-up and habituation period prior to each experimental trial. Both pictures on the right show the SVL estimation procedure. Note the overlapping focal anole and ruler. The corresponding photograph of the ruler has been reduced in transparency and superimposed on the picture of its corresponding individual. Two lines have been drawn to indicate where the vent starts and the snout finishes. In some instances, an arc was subscribed from the anole’s snout to touch the ruler, as if it were lying flat against the tree (right bottom).
(PDF)

S3 Fig Association between the number of perches available for each trial and the actual number of perches used by a lizard in the long-distance trials in Experiment 2 (left; R^2^ = 0.91; p < 0.0001). Data from the short-distance experiment with the same number of mealworms (i.e., five mealworms in Experiment 1) is provided for comparison (right; R^2^ = 0.92; p < 0.0001). Lines in sunflower shaped points indicate the number of individuals with the same values.
(PDF)

S4 Fig Association between the latency to feed from the experimental feeding tray in each experimental trial and the number of perches used in the long-distance trials in Experiment 2 (left; R^2^ = 0.45; p = 0.005) as compared with trials with the same number of mealworms (i.e., five mealworms) from Experiment 1 (right; R^2^ = 0.01; p = 0.68). (PDF)

S5 Fig The relationship between the latency to feed from the experimental feeding tray and the number of perches was similar when there was a direct interaction with a conspecific (right; R^2^ = 0.65; p = 0.11) compared to cases where no conspecifics approached the foraging tray (left; R^2^ = 0.71; p = 0.12). Note, however, that these effects are not significant likely due to the small sample size for each comparison (n = 6 and n = 7, respectively).
(PDF)

S1 Table Mean and range for the values of body size (i.e., SVL), perch height and perch diameter of the focal lizards as well as the temperature and humidity at the beginning of the experiment. We also determined the number of conspecific males within a 7-m radius around the focal lizard at the end of the trial.
(DOCX)
