# Supplementary figures and images for: Predation Risk Perception, Food Density and Conspecific Cues Shape Foraging Decisions in a Tropical Lizard
Source: PLoS One. 2015 Sep 18;10(9):e0138016. doi: 10.1371/journal.pone.0138016 (PMC4575047; doi:10.1371/journal.pone.0138016)

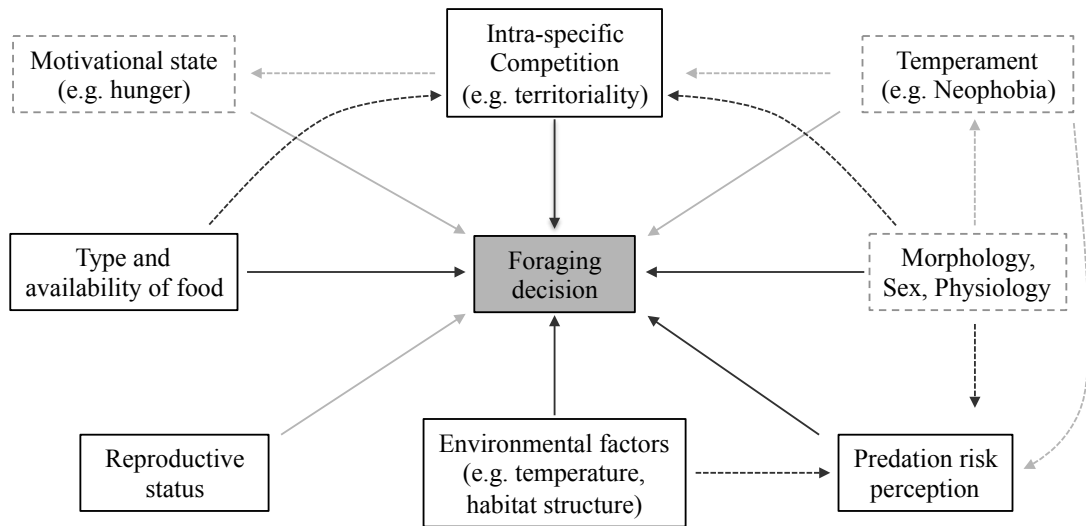

Supplement: S1 Fig — Dashed-line boxes indicate intrinsic factors and solid-line boxes are extrinsic factors. Arrows connecting factors represent the direction of the effects, which are both direct (solid lines) and indirect (dashed lines). Indirect effects influence the foraging decision by modifying an intermediate factor. Factors not assessed in this study are included here to provide a more complete conceptual framework for discussion. (PDF) [file pone.0138016.s001.pdf]

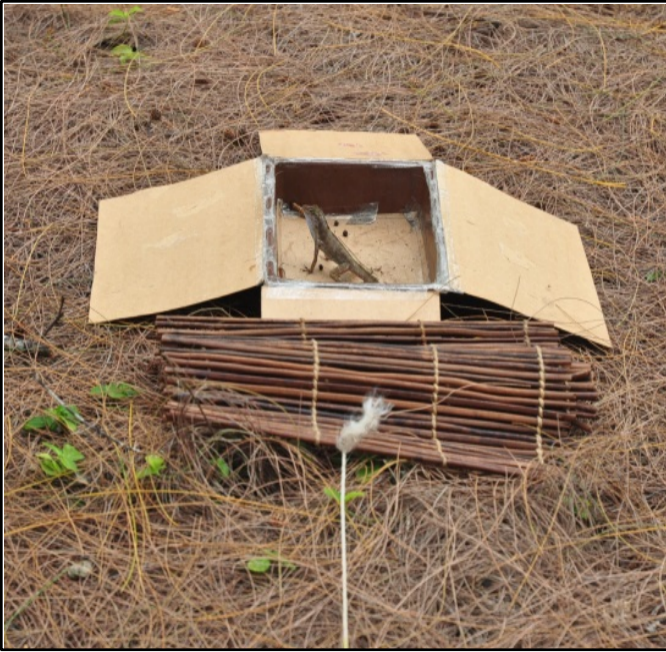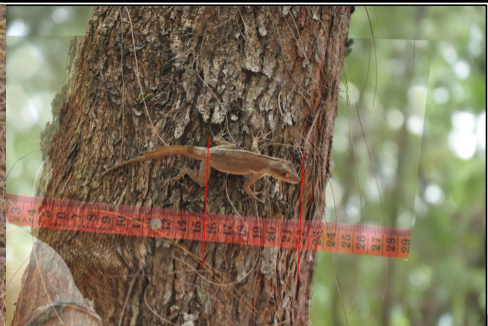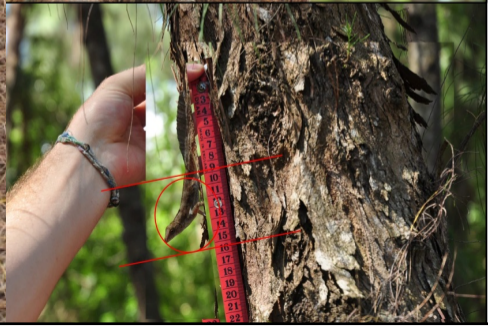

Supplement: S2 Fig — The picture shows a male A. cristatellus consuming a mealworm. Also visible in the foreground is the retracted cover used to hide mealworms from view during the set-up and habituation period prior to each experimental trial. Both pictures on the right show the SVL estimation procedure. Note the overlapping focal anole and ruler. The corresponding photograph of the ruler has been reduced in transparency and superimposed on the picture of its corresponding individual. Two lines have been drawn to indicate where the vent starts and the snout finishes. In some instances, an arc was subscribed from the anole’s snout to touch the ruler, as if it were lying flat against the tree (right bottom). (PDF) [file pone.0138016.s002.pdf]

### Long distance trials

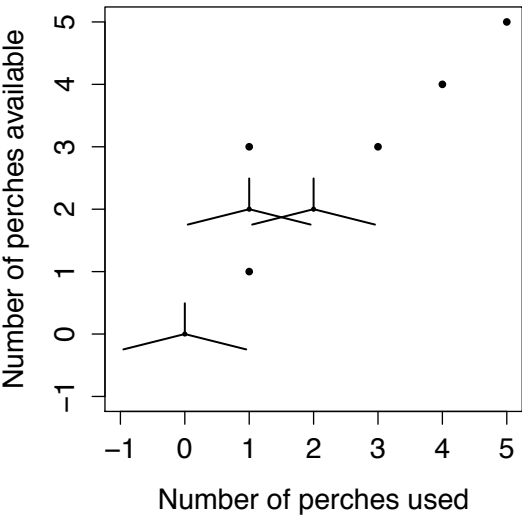

### Short distance trials

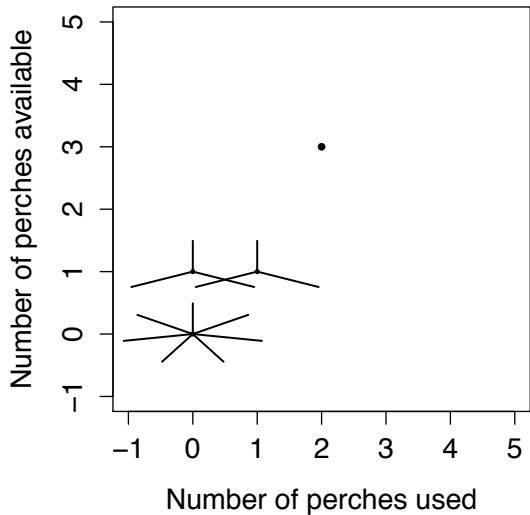

Supplement: S3 Fig — Data from the short-distance experiment with the same number of mealworms (i.e., five mealworms in Experiment 1) is provided for comparison (right; R2 = 0.92; p < 0.0001). Lines in sunflower shaped points indicate the number of individuals with the same values. (PDF) [file pone.0138016.s003.pdf]

**Long distance**

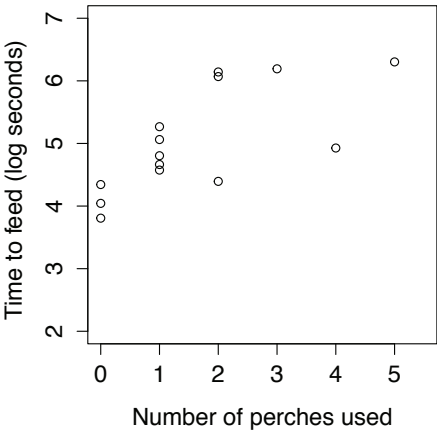

**Short distance**

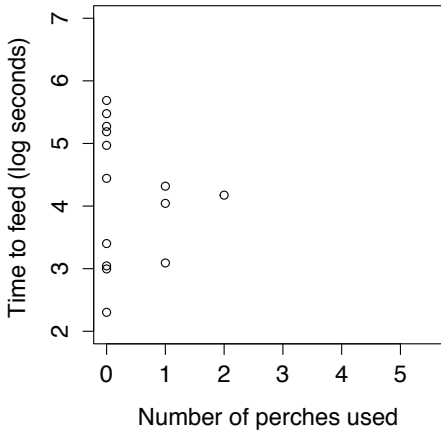

Supplement: S4 Fig — (PDF) [file pone.0138016.s004.pdf]

**Without conspecifics present**

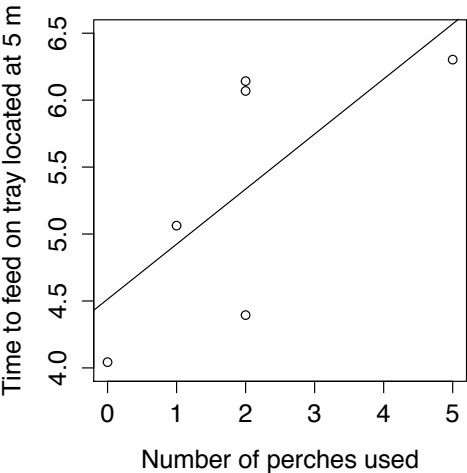

**With conspecifics present**

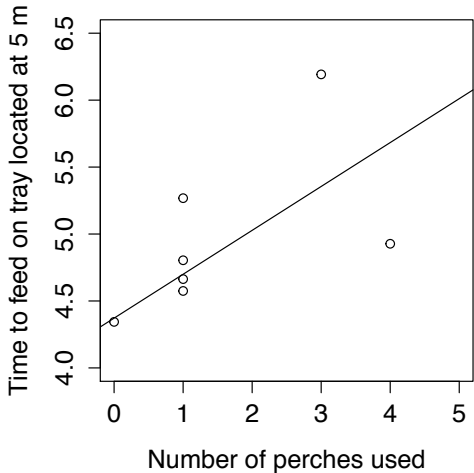

Supplement: S5 Fig — Note, however, that these effects are not significant likely due to the small sample size for each comparison (n = 6 and n = 7, respectively). (PDF) [file pone.0138016.s005.pdf]
