# Supplementary material for: Predation Risk Perception, Food Density and Conspecific Cues Shape Foraging Decisions in a Tropical Lizard
Source: PLoS One. 2015 Sep 18;10(9):e0138016. doi: 10.1371/journal.pone.0138016 (PMC4575047; doi:10.1371/journal.pone.0138016)
Supplement: S1 Table — We also determined the number of conspecific males within a 7-m radius around the focal lizard at the end of the trial. (DOCX) [file pone.0138016.s006.docx]

| Experimental treatment | SVL | Temperature | Humidity | Perch  height | Perch  diameter | Number conspecifics |
| --- | --- | --- | --- | --- | --- | --- |
| Two mealworms | 6 (5.3 - 6.5) | 28.3 (25 - 31) | 58.1 (49 - 67) | 133 (70 - 200) | 44 (4 - 107) | 1 (0 - 5) |
| Five mealworms | 6.2 (5.1 - 7.1) | 28.9 (26 - 32) | 50.3 (44 - 59) | 137 (60 - 232) | 67 (30 - 130) | 1.6 (0 - 4) |
| Ten mealworms | 6.0 (4.9 - 6.7) | 28.4 (26 - 30) | 58.3 (47 - 76) | 123 (66 - 240) | 75 (8 - 142) | 1.8 (0 - 4) |
| Long distance | 6.2 (5.1 - 6.9) | 28.6 (26 - 33) | 50.4 (43 - 59) | 135 (39 - 270) | 69 (32 - 129) | 0.9 (0 - 3) |
